# A methodology for planning, implementation and evaluation of skills intelligence management – results of a design science project in technology organisations

**DOI:** 10.3389/frai.2024.1424924

**Published:** 2024-08-07

**Authors:** Kadri-Liis Kusmin, Peeter Normak, Tobias Ley

**Affiliations:** School of Digital Technologies, Tallinn University, Tallinn, Estonia

**Keywords:** skills intelligence, skills instability, organisation management, organisational communication, evidence-based decision making, decision support system

## Abstract

**Introduction:**

The evolving labour market requirements amidst digital transformation necessitate robust skills intelligence for informed decision-making and adaptability. Novel technologies such as Big Data, Machine Learning, and Artificial Intelligence have significant potential for enhancing skills intelligence.

**Methods:**

This study bridges the gap between theory and practice by designing a novel software artefact for skills intelligence management. With its systematic framework for identifying skills intelligence elements, an assessment instrument, and an implementation methodology, the artefact ensures a thorough approach to skills intelligence management.

**Results:**

The artefact was demonstrated in 11 organisations. Feedback collected from interviews, focus group sessions, and observations (*N* = 19) indicated that the artefact is a feasible starting point for implementing or systematising skills intelligence management. Participants suggested improvements but concurred that the systematic approach enhances skills intelligence data collection and quality.

**Discussion:**

The study shows that the artefact facilitates the application of advanced technologies in skills intelligence management. Additionally, it contributes a set of principles for effective skills intelligence management, fostering a broader conversation on this critical topic. Participants’ feedback underscores the artefact’s potential and provides a basis for further refinement and application in diverse organisational contexts.

## Introduction

The rapidly evolving needs of the labour market place higher demands on workforce skills, and the increased dynamism of the labour market needs to be compensated with more frequent and adaptive upskilling of workers (Cedefop, 2020b). Skills intelligence is the outcome of expert-driven identification, analysis, synthesis and presentation of qualitative or quantitative information about skills and the labour market, drawn from multiple sources and adjusted to the needs of different users (Cedefop, 2019). It is pivotal in informed decision-making (Cedefop, 2020a) to ensure efficient workforce skills management. Skills intelligence helps address skills gaps through quick and automated data analysis for dynamic needs (Cedefop, 2020b; Maldonado-Mariscal et al., 2023). Novel digital technologies, particularly Artificial Intelligence (AI), enhance decision-making for personalised training, recruitment and Human Resources processes, but this requires quality data and collaboration from stakeholders (DiRomualdo et al., 2018; Maldonado-Mariscal et al., 2023). Organisations often need more suitable technology infrastructure and support in data collection and analysis standardisation (Patil and Priya, 2024).

This study seeks to bridge this gap by enhancing the understanding and application of skills intelligence within a single organisation’s context. The current study is one cycle embedded into a more extended study that explores how novel digital technologies can be leveraged in collectively adapting to skills instability. Artefacts generated in the previous cycles form the foundation of the current study. The first study proposed a conceptual data-driven competency management platform to mediate competency-related data between stakeholder organisations to generate skills intelligence (Kusmin et al., 2017). The second study (Kusmin et al., 2018) explored how competency information could be mediated between universities and business organisations to advance workforce skills-related cooperation. The central artefact of the study was a functional software application based on the concept from the first study: a prototypical competency-management platform for universities, business organisations and students. The results of the study suggested that to achieve effective cooperation around workforce competencies, all stakeholders need to go through organisational changes, and thus, it was advised to explore the context of different organisations. The current study is a logical continuation that aims to develop conceptual software further to support the implementation of skills intelligence management practices in business organisations. This reflects a shift towards addressing individual organisations’ unique needs and challenges to realise the benefits of skills intelligence on a more granular level. To achieve this, the following research questions were formulated:

- What are the core elements of skills intelligence management in an organisation?
- How can skills intelligence management be effectively implemented in an organisation?
- How to evaluate the current skills intelligence situation in an organisation?
- What are the core principles for supporting skills intelligence management implementation?

Our paper presents a novel artefact for skills intelligence management, including an implementation methodology and evaluation instrument. We identify challenges related to skills intelligence and explore the concept’s strategic importance beyond human resources. The proposed framework aims to improve skills-related data quality, providing organisations with a foundation for leveraging Big Data (BD) to facilitate the application of novel technologies such as Artificial Intelligence (AI) and Machine Learning (ML) in Human Resources (HR) processes. A significant contribution of the paper is bridging the gap between practice and theory to foster a broader conversation around skills intelligence.

This article is organised as follows. First, we provide an overview of the background, discussing the current skills instability and the subsequent need for enhanced skills intelligence. We explore potential technologies that can improve the skills intelligence landscape and their data processing requirements. We identify the research gap, formulate the research questions, and outline the study’s objectives. Next, we detail the study’s methodology and describe its iterative process. Following this, we present the designed artefact and its contributions to knowledge. Finally, we discuss the implications, limitations, and directions for future research.

Our analysis demonstrates that current skills intelligence practices face significant challenges like sporadic and unstructured data collection. Despite its growing importance, skills intelligence has yet to be widely recognised, indicating a need for broader dissemination and standardisation. Participants confirmed that skills intelligence extends beyond HR, providing strategic advantages. Inter-organisational cooperation emerged as crucial, with shared challenges addressed collaboratively. Implementing a systematic approach to skills intelligence management is essential for its effective application, paving the way for future enhancements through novel technologies.

## Theoretical background

### Skills instability and skills intelligence

Digital transformation has caused significant shifts in the labour market, leading to a rapid worldwide increase in skills instability. New technological advancements cause new skills to emerge, existing skills to evolve, and others to expire due to automation (Gartner Inc., 2020). The COVID-19 pandemic accelerated this pace, making digital technologies imperative for work, learning, and social activities, necessitating digital skills for all jobs (European Commission, 2021; Méndez-Domínguez et al., 2023). To remain competitive, there is an increasing need for constant upskilling and reskilling (Cedefop, 2020b). Life-long learning supports citizens in strengthening their skills throughout life, allowing them to thrive in fast-evolving workplaces (European Commission, 2016).

Organisations require accurate forecasts about future work requirements to make informed decisions about upskilling, training, or recruitment (Bonen and Loree, 2021). CEDEFOP, the European Centre for the Development of Vocational Training, defines skills intelligence as the outcome of an expert-driven process of analysing, synthesising and presenting quantitative or qualitative information on the labour market and skills (Cedefop, 2019). However, the information provided by CEDEFOP is mainly helpful for policymaking and less so for company-specific needs (Maldonado-Mariscal et al., 2023). This article focuses on the organisation-specific aspects of skills intelligence, characterised by higher dynamism for making short-term data-driven HR decisions. With the labour market changing rapidly, there is an urgency to employ novel digital technologies to promote these processes.

### Enhancing skills intelligence management with digital technologies

Digital technologies, including Big Data, Artificial Intelligence, and Machine Learning, transform HR processes by enabling personalised, just-in-time training, predictive workforce planning, and strategic decision-making (DiRomualdo et al., 2018; Patil and Priya, 2024). These advancements in digital tools are integral to enhancing skills intelligence management by providing more detailed data regarding candidate and employee competencies, attitudes, perceptions, and behaviors. This data enables personalised, context-specific training and learning support when combined with other relevant information.

Analytics capabilities help anticipate each individual’s future learning needs and most effective training methods while providing insights into the drivers of effective training and pinpointing methods to generate measurable performance improvements. Despite these advancements, HR analytics faces challenges such as insufficient data quality, standardisation and integration (Angrave et al., 2016; Patil and Priya, 2024). The theoretical models of BD’s contribution to Human Resource Management (HRM) are still scarce in empirical application (Garcia-Arroyo and Osca, 2021). Organisations must understand which data are needed for skills intelligence inferences and their quality requirements to ensure accurate and relevant outcomes.

Research on analytics and BD in HR has mainly revolved around what should be done, but there needs to be more understanding of how it can be done in different contexts and the subsequent results (Angrave et al., 2016). The collaborative nature of skills intelligence demands a collective effort from stakeholders in gathering, analysing, and interpreting data to achieve a nuanced understanding of skills dynamics and enable informed decision-making (Maldonado-Mariscal et al., 2023). Gathering and aggregating skills-related information from different organisations and applying ML approaches can help accelerate the feedback loop between skills intelligence stakeholders (Kusmin et al., 2018).

### Related work

Table 1 provides an overview of the most relevant scientific literature analysed to assess the current knowledge on using novel technologies in HR and improving skills intelligence. The existing literature predominantly focuses on theoretical frameworks, sector-specific applications, and the broad potential benefits of skills intelligence and digital HR transformation. However, empirical studies providing actionable insights and practical guidelines for organisations to integrate these technologies into their HR practices effectively are limited.

**Table 1:** Reviewed literature with focus, main contributions and overview of input to the current research.

Author(s) & year	Focus	Contributions	Input to current research
Angrave et al. (2016)	Critique of the too optimistic outlook on HR analytics, current state and potential drawbacks of HR analytics adoption	Challenges the current perception of HR analytics’ effectiveness and highlights the need for better methods and approaches in HR analytics	The gaps in current HR analytics capabilities
Ruiz-Calleja et al. (2022)	State of the art of Workplace Learning Analytics (WPLA)	Overview of the current state and future directions of WPLA	Overview into data-driven learning and development and the need for broad and multi-source data collection
DiRomualdo et al. (2018)	Impact of digital technology on HR services	Recommendations for transforming HR organisations and improving HR capabilities, service offerings, and performance	Supports the use of digital tools for HR improvement and emphasizes the need for strategic planning and role redefinition
Kusmin et al. (2018)	University-industry partnerships to develop future workforce skills through novel technologies	Framework for enhancing skills intelligence related cooperation through Big Data based continuous feedback	Understanding of the role of stakeholder partnerships in skills development and data-driven decision-making in competency development
Rentzsch and Staneva (2020)	European and international public and private-sector developments for skills intelligence	Overview of skills classification systems and their applications and the potential of combining expert-created and data-driven ontologies	The use of data-driven skills taxonomies for skills intelligence and the need for annotating educational programs with skills taxonomies
Verma et al. (2020)	Improving HR functions in SMEs through Big Data	Overview of potential of BD to enhance HR functions and innovation competency in SMEs and the importance of BD quality for strategic HRM	Exploration of the role of Big Data in HRM and the need for high-quality data to improve HR practices and innovation competency
Garcia-Arroyo and Osca (2021)	Systematisation of academic inputs on Big Data in HR, its implications, challenges and main contributions	Overview of BD in HRM, including five main clusters of HR practice systems where BD is applied, the importance of interdisciplinary studies and practical applications	Insight into the impact of BD in HRM with emphasis on the importance of addressing technical, methodological, and ethical challenges in BD applications
Lalitha Kavya et al. (2023)	Overview of Digital HR publication trends and important themes	Overview of Digital HR literature and key research directions	Aligns with the growing interest in digital HR transformation and highlights its key areas and trends
Maldonado-Mariscal et al. (2023)	Skills intelligence in the steel sector	Reflections on skills intelligence in the steel sector and operationalization of skills intelligence at a sectoral level	Directly addresses the need for company-specific skills intelligence
Méndez-Domínguez et al. (2023)	Access to technologies and digital skills based on socioeconomic characteristics, opportunities offered by new learning environments	Confirmation of the accelerated need for digital skills due to COVID-19 and insights into digital divide and social inclusion	Highlights the urgency of digital skills in the labor market and the importance of addressing digital inequalities
Micheli et al. (2023)	Current knowledge, tools and key factors on Workforce Planning (WFP)	Guidelines for WFP in project-driven companies and identified need for standardised processes and tools in WFP	Understanding of workforce planning in project-driven environments and the importance of standardisation and high-level guidelines in workforce planning
Popo–Olaniyan et al. (2022)	Adoption and impact of AI and analytics on HR decision-making	Insights into the transformative role of AI in HR, overview of potential benefits and challenges of AI-driven talent analytics	Provides insights into AI’s role in transforming HR functions, strategic decision-making and continuous learning
Tammets and Ley (2023)	Integration of AI technologies in (teacher) professional learning, adaptive teaching, professional vision, and decision-making processes	Conceptual model for integrating AI in teacher professional learning	Insight into AI’s impact on workplace learning and the importance of integrating technology into professional development practices
Patil and Priya (2024)	Enhancing strategic business through HR data analytics, implementation issues	Opportunities and challenges of HR data analytics	Emphasizes the importance of HR data analytics in strategic business and the need for data-driven decision-making

The literature overview uncovered the following gaps in research related to skills intelligence:

#### Sector-specific limitations

Many studies, such as those by Rentzsch and Staneva (2020) and Maldonado-Mariscal et al. (2023), focus on sector-specific applications of skills intelligence (e.g., the steel sector, European public and private sectors). These studies do not address the broader applicability of skills intelligence across various industries.

#### Lack of standardisation

Research by Micheli et al. (2023) highlights the need for more standardisation in workforce planning processes, leading to heterogeneous approaches among different organisations. Standardised practices and guidelines that can be universally applied are needed.

#### Implementation challenges

Studies such as those by DiRomualdo et al. (2018) and Patil and Priya (2024) discuss the transformative potential of novel digital technologies in HR but do not provide detailed, empirical evidence on how organisations can overcome implementation challenges, particularly in data collection and analysis standardisation.

#### Focus on theoretical approaches

While reviews like Angrave et al. (2016) and Garcia-Arroyo and Osca (2021) provide critical analyses and theoretical insights, there is a scarcity of empirical data and practical case studies that demonstrate successful implementation of skills intelligence and digital HR practices in real-world settings.

#### Inter-organisational cooperation

Research by Kusmin et al. (2018) suggests that organisational changes are needed to achieve effective cooperation around workforce competencies. However, more insight is needed into how individual organisations can implement these changes to facilitate such cooperation.

The current seeks to bridge these gaps by thoroughly exploring how skills intelligence can be effectively managed and implemented within a single organisation’s context. It is the first to combine theoretical and practical aspects through empirical research and the development of practical tools and methodologies to standardise and successfully implement skills intelligence management across diverse organisational contexts.

## Methods

### Research method

Due to the nature and context of the problem, the study followed design science (DS) research principles. To scaffold the procedures of design science research, the research was guided by the design science research methodology (DSRM) by Peffers et al. (2007). Based on the methodology, the study followed a design and development-centred approach, which entails three activities: design and development, demonstration, and evaluation. By proposing an approach for skills intelligence management, planning and evaluation, this study builds on the earlier works that addressed these challenges and outlined similar solutions for broader application across multiple organisations (Kusmin et al., 2017, 2018, 2019).

### Research activities

**Figure 1:**
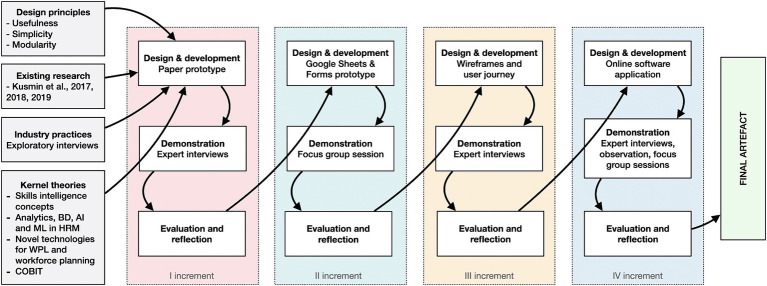
Research inputs, artefact evolution, demonstration methods, and main evaluation focuses of each increment.

The study’s central artefact was developed and improved over four increments. The first increment built on the authors’ previous studies and complemented them with kernel theories on various skills intelligence concepts, analytics, BD, AI, and ML in HRM, novel technologies for WPL and workforce planning, and the more practical COBIT program initiation approach. Figure 1 depicts each increment’s research inputs, artefact evolution, demonstration methods, and primary evaluation focuses.

### Design and development

In the design and development phase, the primary artefact created and iteratively improved was a software application supporting skills intelligence management in an organisation. The artefact was developed by integrating “sub-artefacts”: a framework for mapping skills intelligence management key elements, a methodology for skills intelligence management implementation, and a skills intelligence situation assessment instrument. The software development process followed agile development principles and was, respectively, designed to prioritise usefulness, simplicity and modularity. From a usefulness perspective, the artefact needs to provide immediate benefits to early adopters regardless of the small user base. Thus, the system was somewhat tailored to the needs of the participants. To ensure the fast uptake of the system, another important design aspect was simplicity, which necessitated a strong emphasis on UI/UX considerations. Finally, the system was designed to ensure modularity in both its functional and architectural aspects.

The artefact evolved through four iterations and took several forms: a paper-based prototype, a functional prototype using Google Sheets and Forms, a UI/UX-focused wireframe prototype to specify the user journey, and finally, the completed functional software system.

### Primary artefact: a software platform for skills intelligence management

**Figure 2:**
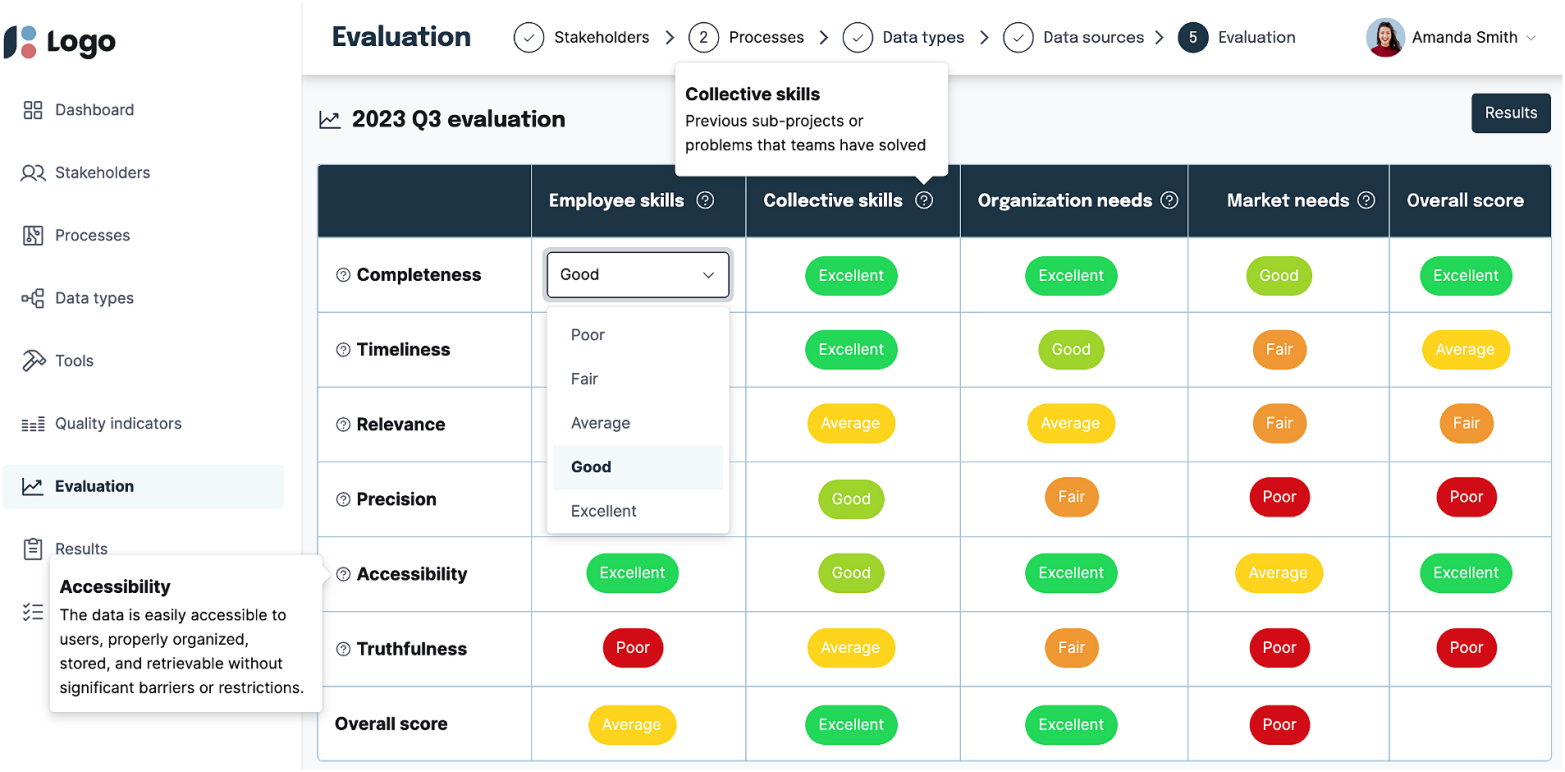
Software artefact visual.

The primary artefact, a software platform, consolidates skills intelligence management into a structured and integrated system, presenting a thorough and transparent process for skills intelligence management activities. After evaluations, the results are summarised and visualised to users. Based on this information, stakeholders can meet to establish targets, agree on future activities and allocate tasks and responsibilities. Users have an overview of activities and statuses on the main dashboard between evaluation periods. Figure 2 is an example of a wireframe of the software platform depicting the evaluation step of the methodology. The source code of the software platform server^1^ and client^2^ are available on public GitHub repositories.

### Sub-artefact: skills intelligence management framework

**Figure 3:**
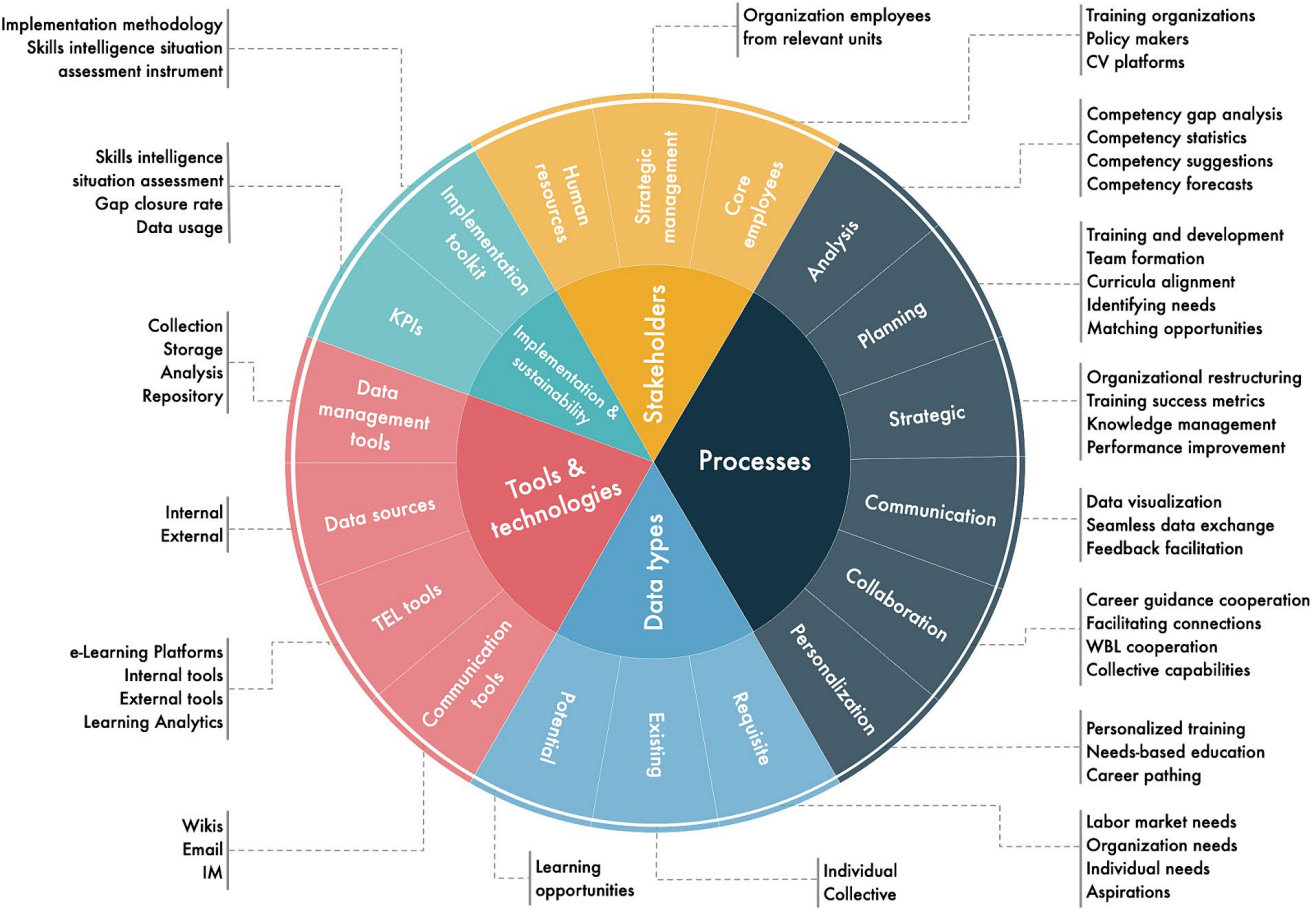
SIMPIE framework.

**Figure 4:**
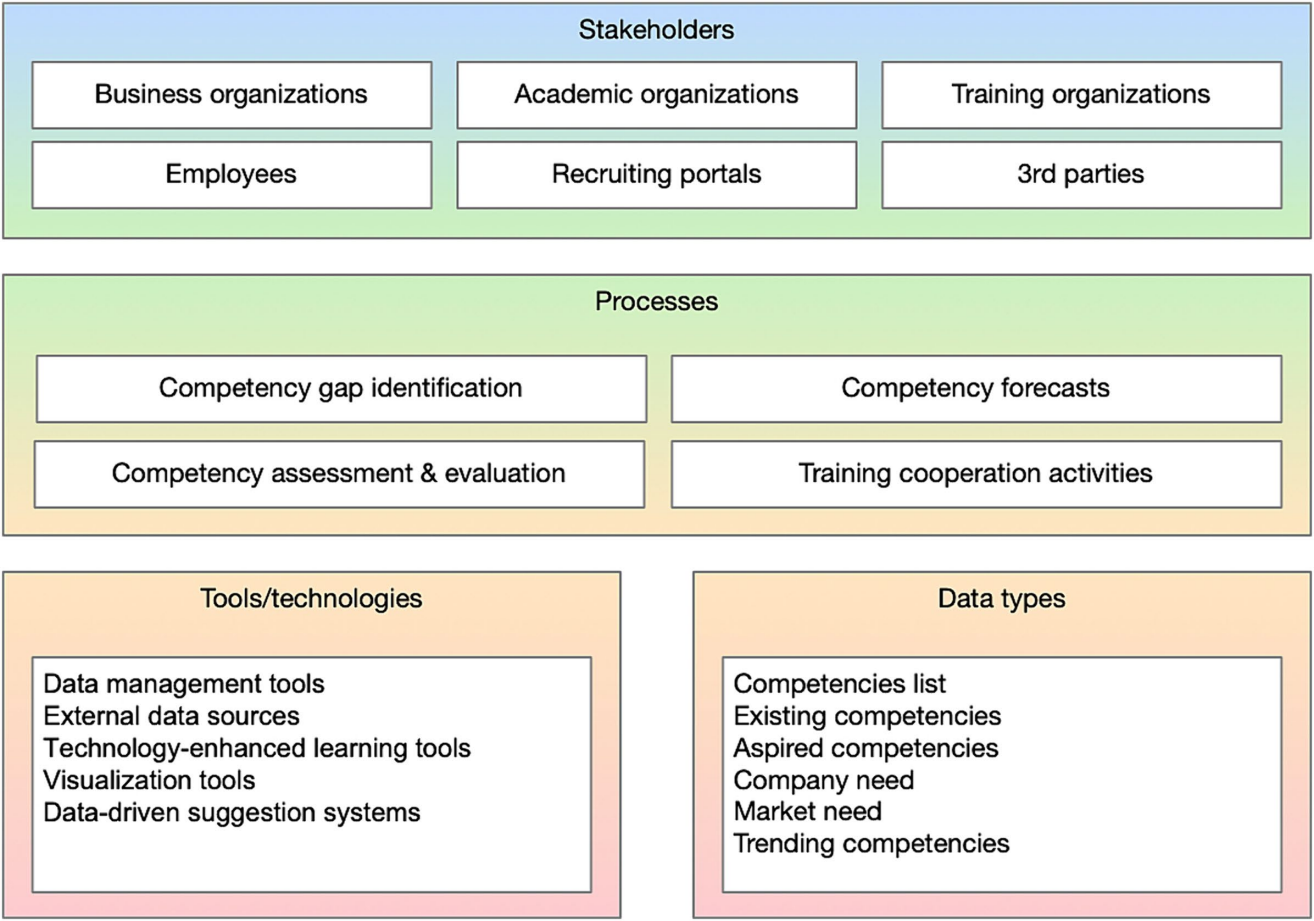
Visualization of the skills intelligence management framework elements adapted from Kusmin et al. (2017, 2018).

The Skills Intelligence Management Planning, Implementation and Evaluation (SIMPIE) framework (depicted in Figure 3) was developed to specify the initial functional requirements of the artefact. This was done by first summarising the findings from Kusmin et al. (2017) and Kusmin et al. (2018), depicted in Figure 4, then mapping the critical elements of skills intelligence management: stakeholders, processes, data types, and tools and technologies and identifying the relationships between these elements. It is important to note that the elements in the framework serve as a reference and are not intended to be a comprehensive collection. Visualising the framework elements is primarily intended for the initial SIMPIE activities, serving as a template and guidance. However, it also acts as a visual reference when onboarding new participants to skills intelligence management processes.

### Sub-artefact: skills intelligence situation assessment instrument

The skills intelligence situation assessment instrument was developed to help organisations understand their skills intelligence-related data priorities and current data quality. The instrument provides a clear list of activities all participants must follow in skills intelligence management processes. As the first assessment activity, the participants list the activities (processes) that depend on skills intelligence and map the data types used in these processes. The participants identify the data source(s) and their respective supervisors for each data type. Each of the data types is then assessed against previously agreed quality criteria. This results in a matrix of quality criteria, data types, their scores, and feedback to data source supervisors. During the assessment process, participants are requested to evaluate each data type against the quality criteria on a Likert scale ranging from 1 (poor) to 5 (excellent). The matrix provides two sets of skills intelligence inferences based on the combined assessment results. First, it calculates the average quality score for each criterion across all examined data types. This represents how well the data meets specific quality aspects, such as how complete the data is on average. Secondly, it provides an overall quality score for each data type, representing its strengths and weaknesses across multiple criteria, e.g., completeness, timeliness and accessibility.

**Figure 5:**
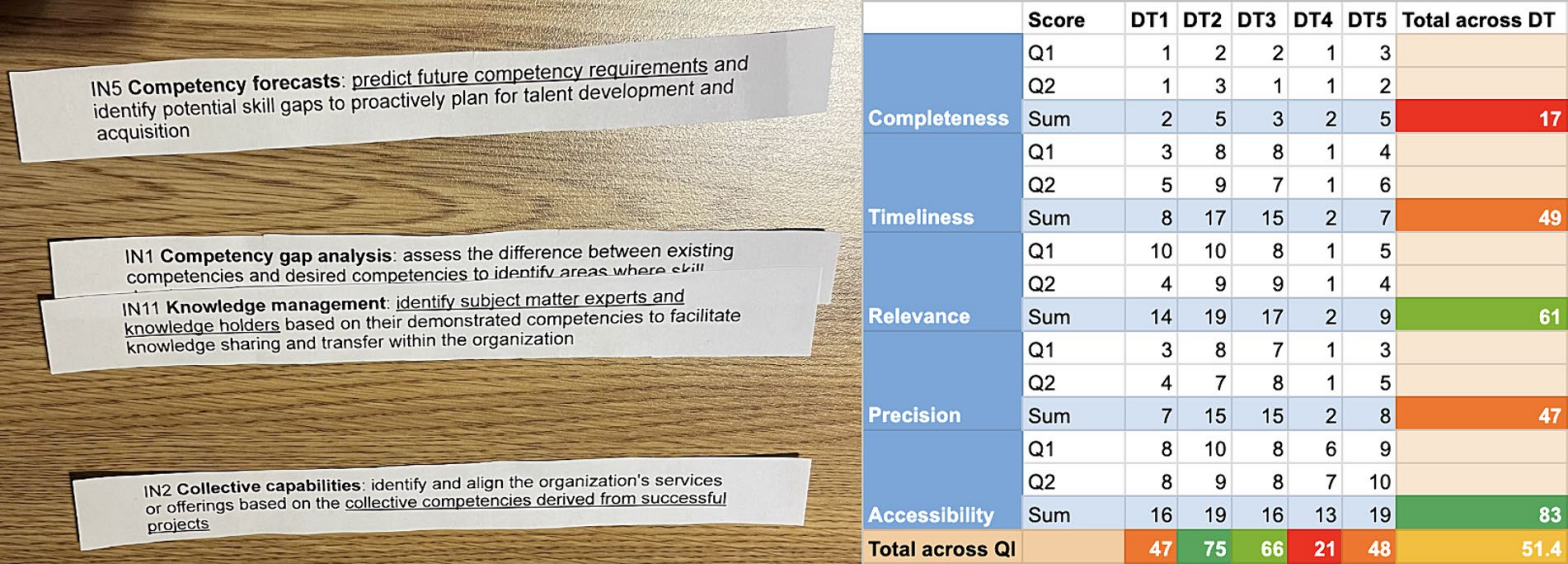
Prototypes of skills intelligence situation evaluation instrument.

The organisation can plan and prioritise the actions needed to improve skills intelligence based on the results. Lower data type quality scores indicate a need to redesign the process for collecting, storing, and communicating this data type and include the stakeholders who prioritised this data type higher. Low scores across a particular quality criterion indicate more technical problems: for example, the data collection frequency is low (timeliness), or the established data models are too vague for their intended purposes (precision). Additionally, the results will serve as a baseline for the subsequent evaluation to calculate the KPIs. The prototypes of the instrument can be seen in Figure 5.

### Sub-artefact: skills intelligence management implementation methodology

**Figure 6:**
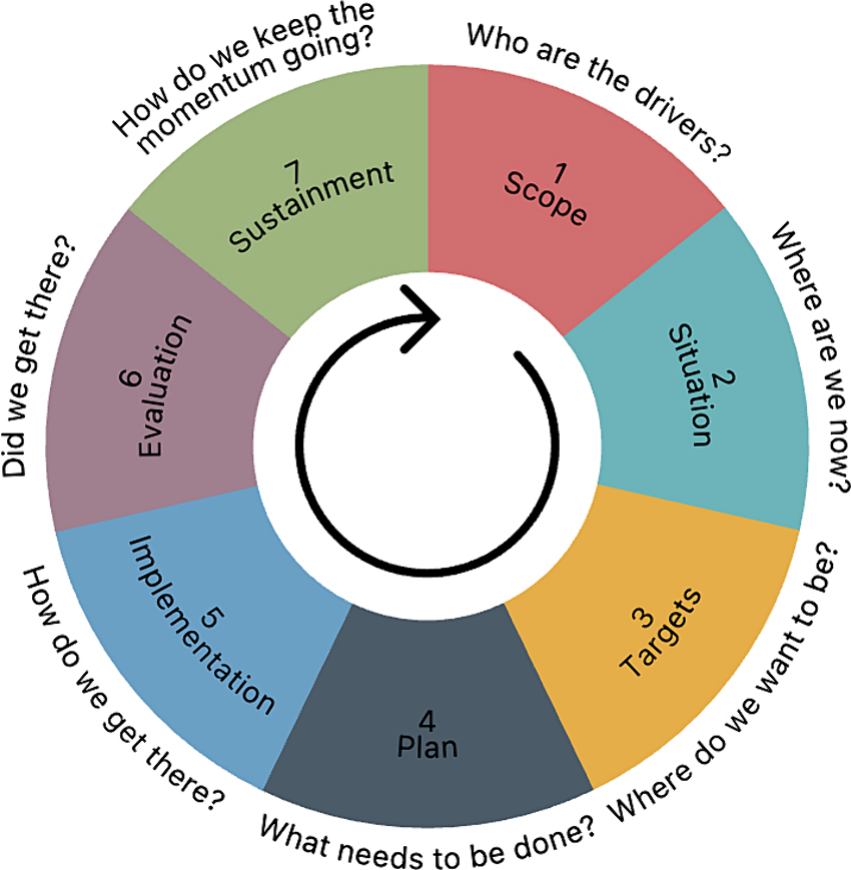
SIMPIE implementation methodology.

The platform functionalities were incrementally combined into a comprehensive methodology based on the COBIT program initiation approach phases (ISACA, 2018). Using the COBIT system, where each implementation phase must answer a particular question, seven similar phases were mapped: scope, situation, targets, plan, implementation, evaluation, and sustainment. The implementation phases are depicted in Figure 6.

**Phase 1, “Scope”,** corresponds to the COBIT program initiation phase that answers the question “What are the drivers?” However, the Scope asks, “Who are the drivers?” This phase consists of stakeholder identification and scope definition, which can be done through simple open-ended interviews. During the interview, the participants analyse the generic framework structure and map the most critical elements in the organisation’s context. The interview can serve as a tool for snowball sampling as participants can suggest other people who should be included in the process.

**Phase 2, “Situation”,** corresponds to the COBIT problems and opportunities definition phase. It answers the question, “Where are we now?”. The first activity of this phase is aligning specific organisation details with the corresponding framework elements. For instance, the framework element “stakeholders” could include “strategic management,” “HR,” or even more specifically, an actual person’s name. Similarly, the process “competency suggestions” could be “provide personal suggestions for horizontal career development opportunities.” The second activity of the phase is skills intelligence evaluation, which is conducted with the skills intelligence evaluation instrument.

**Phase 3, “Targets”,** outlines the expected outcomes of the process and answers the question “Where do we want to be?” The first action is to analyse the outcomes of the skills intelligence matrix to prioritise the data types and quality indicators that need to be improved and establish the expected values for the subsequent evaluation. The second activity is to specify and prioritise actions. The suggested form for this activity is focus group sessions.

**Phase 4, “Plan”,** answers “What needs to be done?”. The results of phase 3 are represented as scenarios, which, in turn, are presented as an IS architecture to outline its development plan. The suggested form for this phase is collaborative design sessions.

**Phase 5, “Implementation”,** answers the question “How do we get there?” and is devoted to developing and implementing processes and information systems. This phase differs from the previous phases in that it should follow the principles of software development projects. This means that the Implementation phase might be iterative.

**Phase 6, “Evaluation”** (“Did we get there?”), is a follow-up evaluation via the skills intelligence evaluation instrument. The results are analysed, discussed and communicated to the stakeholders. This phase can be carried out individually or in groups.

**Phase 7, “Sustainment”,** seeks to answer the question, “How do we keep the momentum going?” Based on the results of the previous phases, stakeholders’ feedback is collected via interviews, surveys, or other methods. These should be categorised, combined with the KPIs, and phrased as activities to improve the framework implementation.

### Demonstration

As the artefact design and development activities followed agile principles, there were several demonstration points. This was done to minimise risks through a faster feedback loop: the artefact was incrementally improved, and each improvement was demonstrated to make minor adjustments based on participant feedback. The artefact was demonstrated in four key phases: a paper-based prototype testing the methodology that formed the functional requirements of the artefact, a Google Sheets and Forms-based functional prototype, wireframes depicting the user journey through the software artefact, and the final functional software. The first two prototypes can be seen in 6. The final software was deployed online and was publicly accessible, and the source code for both the client and server side was uploaded to public repositories in GitHub.

The initial sample was a convenience sample (Teddlie and Yu, 2007) in a project-based IT organisation, which was expanded through snowball sampling *(ibid)*. In total, the participants (*N* = 19) originated from 11 organisations: a project-based IT software development service provider, a software product provider, the IT departments of two energetics organisations, a retail chain, one government IT organisation, a change management organisation, an international technology organisation and three IT startups. One organisation declined to participate because the shared information would be too sensitive.

The solution’s objectives were qualitative and sought to describe how the new artefact is expected to support the novel problem (Peffers et al., 2007). Thus, qualitative methods were used, including unstructured interviews (preliminary, pre-design), semi-structured interviews (increments I, III, IV), a focus group session (increment II), and observation (increment IV). Preliminary interviews confirmed previous research and explored the first participating organisation. Participants (*N* = 5) described their activities related to skills intelligence, discussed data types and processes, and helped identify additional participants through snowball sampling.

The paper prototype was tested through expert interviews divided into three phases: introducing the initial framework and methodology, assessing the skills intelligence situation using paper cards, and answering survey questions on data quality. Quality scores were calculated, followed by a discussion of the procedure’s feasibility and usefulness.

The second artefact version, a Google Sheets and Forms prototype, was tested in a focus group session with four initial and two new participants (*N* = 6) from the same organisation. The session mirrored the expert interviews, including discussions on skills intelligence, a self-assessment form, and the improved assessment process using Google tools. Participants completed another self-assessment to gain insight into changes in their perceptions.

The third artefact version, consisting of wireframes for a skills intelligence management platform, focused on usability and effectiveness. Expert interviews with two previous and four new participants (*N* = 6) from different organisations evaluated the concept’s feasibility, identified UI/UX and other shortcomings, and suggested improvements.

The final functional software version was demonstrated through expert interviews or focus group sessions with two original and seven new participants (*N* = 9). In this phase, all demonstrations were organised over Microsoft Teams, and the participant(s) or the researcher shared the screen alternately. Participants used the software while the researcher observed, noting navigation times, confusing parts, and missing functionalities. In one case, a technical issue required screen sharing, with the researcher following the participant’s commands. At the end of the session, the participants answered questions addressing the artefact’s feasibility, effectiveness, usability and functionality.

### Evaluation

The demonstration aimed to gather feedback to evaluate various aspects of the artefact, including feasibility, effectiveness, usability, and functionality, and to collect suggestions for improvement. Participants were asked targeted questions to explore their perception of the artefact.

Feasibility:

○ Participants discussed whether implementing the system in their organisation would be possible and how difficult it would be.
○ They described the benefits, obstacles, and challenges of implementation.
○ They reflected on the availability of resources (time, money, and people) in their organisation.

Usability:

○ Participants were asked about the intuitiveness and simplicity of the system.
○ They discussed the learning curve and whether they could use the system independently without the researcher’s guidance.
○ They described the challenges and misunderstandings encountered while using the system.

Effectiveness:

○ Participants evaluated whether the system solves the stated problems and how well it does so.
○ They addressed whether the system could help them achieve their goals better.
○ They were asked if they knew of any better analogues.
○ Functionality:
○ Participants discussed the functionalities they expected from the system.
○ They evaluated whether the system performed as anticipated.
○ They reported any errors they encountered.

Participants’ discussions and reflections on these areas highlighted both the strengths and areas for system improvement. The targeted questions helped us understand their perceptions of refining and enhancing the artefact. The following sections delve into an in-depth system evaluation, informed by the participants’ detailed feedback.

### Feasibility

Participants were generally willing to use the system if the benefits were clear. Challenges included potential resistance and the need for training. Having a designated person in charge of skills intelligence management was seen as a crucial requirement for effective implementation. The system was considered less valuable in organisations where managers have fewer employees, and in two cases (a government organisation and one startup), it was not considered beneficial. Larger organisations are actively exploring potential applications of AI and ML in competency and HR-related practices, with the largest organisations already implementing these technologies. Participants from these organisations strongly agreed on the topic’s urgency and considered the artefact and methodology feasible stepping stones toward further advancements.

The potential for effective application was perceived to be higher if integrated with in-house tools: participants were worried that otherwise, the employees would have yet another piece of software where they would have to create accounts and remember their passwords.

In project-based organisations, strategic management, business unit leads, and salespeople showed more interest than HR or training managers due to their potential to increase the transparency of the organisation’s collective skillset. Many participants stressed that the system must comply with data privacy regulations and the European Union’s General Data Protection Regulation (GDPR) to be adopted. Feasibility evaluation results are summarised in Table 2.

**Table 2:** Feasibility evaluation summary.

Problem	Suggestion	Justification	Limitations
Less urgent need for the system in organisations with fewer employees to manage	Formulate specific audience groups	Prioritise high-need organisations	May overlook smaller-scale implementation benefits
Potential resistance and unclear benefits hinder system adoption	Address potential resistance through training and clear communication of benefits	Measure acceptance and potential usage	May not capture all resistance factors
Resistance and need for training are significant challenges	Address potential resistance through training and clear communication of benefits	Identify barriers and address them	Requires sustained training efforts
Lack of designated person in charge of skills intelligence management	Ensure a designated skills intelligence manager	Ensure dedicated management for system success	Additional organisational resource requirements
“Language barriers” due to inconsistent understanding of terminology	Organize a workshop to standardise terminology and prevent language barriers	Facilitate common understanding and terminology	Requires consensus and participation
Higher perceived feasibility if integrated with in-house tools	Integrate the system with in-house tools like JIRA	Enhance system adoption through familiar tools	Technical challenges in integration
Interest in the system is higher among one group of employees	Highlight benefits for various positions and units	Identify key stakeholders and their needs	May require tailored communication strategies

### Effectiveness

From the perspective of increasing skills intelligence in the organisation, the artefact was considered more effective in organisations with higher skills variability, which may be explained by the fact that higher skills variability leads to greater skills instability. On the other hand, participants from organisations with more firmly established technology and competency requirements found the artefact could have been more effective. This may be attributed to their lack of exposure to skills instability.

After the skills intelligence situation assessment, most participants felt better informed about increasing skills intelligence and the general situation in the organisation. However, their ability to systematically list skills intelligence-related activities decreased. The reasoning might be that they needed to be fully aware of the complexity of the topic prior to the assessment. A marginal improvement was reported in insight into colleagues’ skills in intelligence-related activities. To improve effectiveness, participants suggested including qualitative insights to supplement numerical data. Participants agreed that the system should be highly configurable. Multiple recommendations were made about the base information, e.g., processes, data types, and quality criteria.

From the broader perspective of reducing skills instability overall, participants were concerned that convincing organisations to share their statistics with other skills intelligence stakeholders could be challenging. It was suggested that incentives be explored to persuade organisations to contribute. Table 3 provides an overview of effectiveness evaluation results.

**Table 3:** Effectiveness evaluation summary.

Problem	Suggestion	Justification	Limitations
Lack of qualitative insights to supplement numerical data	Include free-form fields for qualitative insights	Provide a holistic view of effectiveness	Qualitative data can be subjective and harder to analyse
Participants feel uninformed about how to systematically list and manage skills intelligence-related activities	Provide thorough base data	Improve user capability and understanding	May not fully represent all options
Realization of the complexity and depth of skills intelligence-related data types and processes decreases participants’ confidence	Provide support to help participants systematically list and manage skills intelligence-related activities	Address complexity and enhance user skills	May require significant training resources
Limited insight into colleagues’ skills intelligence-related activities	Enhance visibility and sharing of colleagues’ skills intelligence-related activities to improve collective insight	Foster better teamwork and transparency	Privacy concerns and data sharing limitations
Need for additional recruitment metrics in the data types	Include recruitment metrics (e.g., time to hire, cost to hire) as additional data types	Address practical recruitment needs	Additional data collection burden

### Usability

Usability was the most addressed topic during the interviews, and participants suggested many ideas for improvement. Some of these suggestions were contradictory. For example, while some participants found the skills intelligence situation assessment scoring matrix too bothersome and preferred to insert scores using a questionnaire, others felt that this would make the process too long and not provide a clear overview of all the scores they submitted. One participant recommended using gamification and sliders for scoring.

Participants suggested providing more detailed score descriptions and agreeing on definitions and terms. They also recommended integrating the system with existing tools or implementing single sign-on (SSO). Additionally, they proposed offering pre-made templates for a more straightforward setup. Most participants saw a need for a workshop to standardise terminology to avoid nomenclature barriers. Lastly, to avoid language barriers and promote inclusivity, it was suggested that localisation be implemented for users who need to be more fluent in English. Usability evaluation results are listed in Table 4.

**Table 4:** Usability evaluation summary.

Problem	Suggestion	Justification	Limitations
Lack of clarity in score descriptions	Provide detailed descriptions for numeric scores	Capture user satisfaction and ease of use	May not fully represent all user experiences
Inconsistent definitions and terms among users	Establish agreement on definitions	Facilitate common understanding and terminology	Require consensus, which can be difficult to achieve
Setup process is not user-friendly	Create pre-made templates	Simplify the user setup process	Templates may not fit all user needs
“Language barriers” due to varied terminology among participants	Organize workshops to standardise terminology	Ensure consistent terminology use	Time-consuming and requires participation
Potential issues with the uptake of new software	Integrate with existing tools	Ensure seamless workflow with existing tools	Technical integration challenges
Need to create additional user accounts to the new system	Implement SSO	Ensure seamless workflow with existing tools	Technical integration challenges
Interface is only in English	Implement localization	Make the system more inclusive	Technical challenges and translation inconsistencies

### Functionality

The most prominently reported inconsistency regarding functionality was the need for actual skills intelligence data collection functionality in the artefact. While participants understood that the framework and methodology focus on generating meta-data concerning skills intelligence-related data, many expected to be able to use the same platform for both purposes. Multiple participants stated that the platform should encompass all methodology phases, including target-setting, implementation, and sustainment. Additionally, they suggested including a dashboard to visualise all evaluation results compared to the defined targets. This feature would provide evidence of improvement and, in turn, increase motivation. Lastly, it was suggested that the platform should include a recommendations engine to assist with the roadmap in the planning phase. Based on the data types and quality criteria combinations in the situation assessment report, the system could use ML or AI principles to provide a clear plan for the organisation to improve their skills and intelligence.

In addition to contextual functionality evaluation, two expert interviews were conducted to evaluate the technological feasibility of the software. The application was subjected to black box testing to reveal inaccuracies or missing functioning in the software, errors in performance or data structures, and shortcomings in user experience. The interviews lasted one hour and were carried out by a senior quality engineer and a senior software engineer. The functionality evaluation results are summarised in Table 5.

**Table 5:** Functionality evaluation summary.

Problem	Suggestion	Justification	Limitations
Lack of skills intelligence data collection in the artifact’s functionalities	Expectations management	Highlight the intended purpose of the system	The functionality of the system might remain too narrow for some organisations
Not all methodology phases are reflected in software	Add functionalities for target-setting, implementation tracking, and sustainment activities	Ensure the contextual integrity of the system	Adds technical complexity
Potential inaccuracies or missing functionality in the software, errors in performance or data structures, and shortcomings in user experience	Address issues identified through black box testing and expert interviews	Ensure software accuracy, performance, and user experience	Limited to the expertise of the interviewed professionals and the scope of the testing

## Results and discussion

### Skills intelligence management principles

The study contributed to knowledge by designing and developing a series of artefacts that addressed the research questions, which helped map the core principles for implementing skills intelligence management. The study identified five key principle categories for effectively implementing skills intelligence management: illustrate, clarify, tailor, support, and sustain, as illustrated in Table 6.

**Table 6:** Skills intelligence management principles.

Principle Category	Purpose	Principles
Illustrate	Engage stakeholders, gain their interest, and serve as guidelines through effective visualization and illustrative examples.	Engaging examples: capture interest with relatable and engaging illustrations. Templates as guidelines: provide templates or examples for practical guidance. Visualized status: showcase progress landmarks. Visualized targets: provide visualizations of future milestones.
Clarify	Foster a shared and accurate understanding across all skills intelligence management aspects.	Comprehensible evaluation: use word-based scores aligned with respective sets of statements regarding skills intelligence situations. Real-time status: communicate the latest progress. Clear process overview and status: present a transparent overview of process steps. Consensus on terminology: establish agreement on terms, definitions, and meanings. Clear targets: set explicit, unambiguous, and measurable goals. KPIs: uphold clear key performance indicators for accuracy.
Tailor	Ensure dynamism and customizability.	Dynamic configuration: foster adaptability through configuration and customization. Collective implementation: include various stakeholders to integrate the methodology into organisational processes. Configuration workshop: incorporate workshops for collective configuration of key elements (e.g., data types, quality, and evaluation criteria).
Support	Offer essential support structures.	Leverage technologies: support processes with user-friendly and accessible technologies. Integrations: allow integrations with tools like Atlassian JIRA or in-house skills intelligence tools. User experience: ensure usability through simple design and user journey. Suggestions: provide suggestions to guide and train users. Encouragement: foster engagement through encouraging communication.
Sustain	Maintain relevance and currency.	Sustainment practices: collect feedback and organize retrospectives for improvement. Feedback mechanism: enable submission of qualitative feedback to data source administrators in case of negative scores or score combinations. Efficient process: prioritise shorter processes using online tools. Notifications: implement a reliable communication system to keep users informed.

Most of these principles emerged as pervasive themes across the sub-artefacts. However, their application should consider the context: e.g. the subsequent activities differ when implementing skills intelligence management in general or developing supporting applications.

### Other findings

Participants’ feedback confirmed the research problem’s relevance, highlighting that organisations face numerous challenges related to skills intelligence. One significant finding is that the term “skills intelligence” is not widely used in organisations – participants needed an explanation of the term. However, after being provided with examples, all participants reported that they understood the concept and encountered it daily, although under different names.

Participants affirmed that the importance of skills intelligence extends beyond the HR department. Insight into skills demand and supply is considered a strategic advantage for organisations. They agreed that inter-organisational cooperation in this area is increasingly vital. On several occasions, participants were surprised to learn that their coworkers had faced similar skills and intelligence challenges, which could be solved through cooperation.

Most participants acknowledged encountering skills intelligence challenges to varying degrees. While some viewed these challenges as minor inconveniences, others saw significant opportunities for improvement and had initiated plans to address potential solutions. Most agreed that a systematic approach to skills intelligence management would enhance its application on a broader scale within and across organisations. Establishing a formalised structure for collecting skills data would benefit the collective by laying a solid foundation for advanced statistics, forecasts, and other machine learning opportunities. Designated structures for improving skills intelligence also unify vocabulary and expand the term’s adoption. However, there are concerns regarding the broader scale of the research problem—reducing skills instability through advanced skills intelligence across labour market stakeholders. Participants were hesitant when asked whether their organisations would agree to share statistics from their improved skills and intelligence. It was recommended that possible incentives be explored to encourage them to agree.

### Industrial implications

Standardising “skills intelligence” can facilitate better understanding and communication within and across organisations. The study confirms that skills intelligence is not just an HR concern but holds strategic importance for the entire organisation. Insight into skills demand and supply provides a critical market advantage, and participants agreed that inter-organisational cooperation in this area is becoming increasingly important. However, a broader conversation on the topic is necessary, as its importance has yet to be widely recognised.

Implementing the proposed framework could enhance organisational effectiveness and improve employee well-being by taking the first step towards high-quality skills intelligence in Big Data to enable evidence-based decision-making in workplace learning and organisational strategy. Effective implementation of the framework and its software platform offers opportunities for higher quality and more standardised data exchange and communication between organisations. This promotes collaboration among skills intelligence stakeholders to support lifelong learning.

Overall, this study not only contributes to knowledge generation but also has the potential to make a significant contribution to reducing skills instability by equipping organisations with practical tools to adapt to the changing landscape skills in the modern workplace by moving towards high-quality big data and employing novel technologies such as ML and AI for evidence-based decision making.

### Limitations and future work

It is essential to acknowledge the limitations of the study. First, the study was constrained by a small sample size, which might limit the robustness and generalizability of the findings. Additionally, the absence of quantitative evaluation means that the outcomes rely heavily on qualitative assessments, which can introduce a degree of subjectivity. The generalizability and transferability of the results to different contexts has yet to be tested, as the research was primarily conducted within a few organisations, predominantly in the IT sector. This narrow focus raises the need to explore the broader applicability of the findings. A longitudinal study is necessary to fully understand the effects, benefits, and challenges associated with implementing the framework. Such an approach would provide valuable insights into how the framework performs over time and across various organisational contexts.

However, these limitations open new lines for future research. One potential area of inquiry involves applying and evaluating the framework and methodology in diverse organisational contexts across different domains, countries, and cultures. Additionally, while the current study relied primarily on expert interviews for evaluation and validation, a longitudinal study examining the framework’s application within an organisation could offer valuable insights into the dynamics and evolution of its implementation. From a practical standpoint, developing software artefacts using different technologies focusing on innovative human-computer interaction approaches could yield interesting results. One of the more interesting suggestions by the study participants was to include a recommendations engine that would apply ML or AI principles to the gathered meta-data and provide a roadmap for skills intelligence improvement or implementation. Finally, this research highlights that realising the full potential of skills intelligence in the labour market depends heavily on stakeholder cooperation. This presents an opportunity to investigate how data exchange and communication between organisations evolve after implementing effective skills intelligence management systems.

## Conclusion

This paper has presented an artefact for skills intelligence management planning and evaluation in organisations, complete with an implementation methodology, skills intelligence situation evaluation instrument, and implementation principles. By drawing on participant feedback and an extensive review of current practices, we have developed a nuanced understanding of the challenges and opportunities for enhancing skills intelligence in the modern workplace.

Our analysis has highlighted several challenges organisations face regarding skills intelligence, including the sporadic and unstructured collection of skills-related data. Despite the increasing importance of skills intelligence for workforce adaptability, the term itself needs to be more widely recognised, indicating a need for broader dissemination and standardisation.

Participants affirmed that skills intelligence extends beyond HR, offering a strategic advantage. Inter-organisational cooperation emerged as a crucial factor, as many discovered that shared challenges could be addressed collaboratively. A systematic approach to skills intelligence management was essential for its effective application.

We have examined the current state of skills intelligence data collection and identified significant gaps and limitations. To address these, we recommend implementing a comprehensive framework that leverages high-quality skills intelligence Big Data for evidence-based decision-making. This would enhance organisational effectiveness and employee well-being by promoting standardised data exchange and communication.

Our study’s findings suggest that future research should apply the framework across diverse contexts to assess its generalizability, including longitudinal studies and exploring novel technologies for skills intelligence improvement. Developing software artefacts with innovative human-computer interaction approaches could yield interesting results. Participants suggested including a recommendation engine that applies ML or AI principles to the gathered meta-data to enhance skills intelligence.

However, concerns exist regarding the broader scale of reducing skills instability through advanced skills intelligence across labour market stakeholders. Participants were hesitant about sharing statistics generated from improved skills intelligence, indicating a need to explore incentives for encouraging such cooperation.

In conclusion, this research underscores the strategic importance of skills intelligence and highlights the necessity of stakeholder cooperation for its full realisation. While limitations such as a small sample size and a focus on the IT sector suggest further research, this study provides a foundation for future work to adapt to the changing skills landscape. By promoting a proactive, interdisciplinary approach and fostering greater collaboration, we can work towards a future where skills intelligence supports lifelong learning and organisational strategy.

## Data availability statement

The datasets presented in this article are not readily available because the data is qualitative and very case-specific, concerning existing situations and attitudes in business organisations. The participants were ensured that the raw data will not be published. Requests to access the datasets should be directed to K-LK, kusmin@tlu.ee.

## Ethics statement

Ethical approval was not required for the studies involving humans because the research did not include any vulnerable populations. The data collected for the study did not contain any sensitive data and the study posed very low risk for the participants. The studies were conducted in accordance with the local legislation and institutional requirements. Written informed consent for participation was not required from the participants or the participants’ legal guardians/next of kin in accordance with the national legislation and institutional requirements because the study was non-invasive, and the interviews and observations were carried out in a relaxed and familiar situation to the participants, to ensure that their feedback was natural. Verbal consent was acquired.

## Author contributions

K-LK: Conceptualization, Data curation, Formal analysis, Funding acquisition, Investigation, Methodology, Project administration, Resources, Software, Supervision, Validation, Visualization, Writing – original draft, Writing – review & editing. PN: Conceptualization, Supervision, Writing – original draft. TL: Conceptualization, Supervision, Writing – original draft.

## Footnotes

^1^ https://github.com/duttiv/simple-server-public

^2^ https://github.com/duttiv/simple-client-public
